# Nursing practices and complications associated with peripheral catheterization for outpatient antineoplastic therapy: scoping review

**DOI:** 10.1590/0034-7167-2024-0528

**Published:** 2025-12-08

**Authors:** Vanessa Albuquerque Alvim de Paula, Vilanice Alves de Araújo Püschel, Kelli Borges dos Santos, Fábio da Costa Carbogim, André Luiz Silva Alvim

**Affiliations:** I Universidade Federal de Juiz de Fora. Juiz de Fora, Minas Gerais, Brazil; II Universidade de São Paulo. São Paulo, São Paulo, Brazil; III Centro Brazileiro para o Cuidado à Saúde em Evidências: Centro de Excelência do JBI (JBI Brazil). São Paulo, São Paulo, Brazil

**Keywords:** Catheterization, Peripheral, Antineoplastic Agents, Ambulatory Care, Nursing, Complications., Cateterismo Periférico, Antineoplásicos, Atención Ambulatoria, Enfermería, Complicaciones.

## Abstract

**Objectives:**

to map the evidence on nursing practices related to outpatient antineoplastic therapy using peripheral intravenous catheters and to identify the complications associated with the use of this device.

**Methods:**

this is a scoping review conducted according to the JBI methodology. The selection process was carried out independently and blindly by two reviewers.

**Results:**

a total of 1,287 publications were retrieved, and 16 studies were ultimately included. The identified practices were related to responsibility for the procedure; infection prevention and biosafety; selection of the puncture site; choice of the puncture device; skin preparation; venous puncture; access maintenance; catheter dressing and stabilization; and device removal and disposal. The most frequently reported complications were phlebitis, edema, pain, burning sensation, extravasation, and bloodstream infection.

**Conclusions:**

this study mapped peripheral intravenous catheterization practices for outpatient antineoplastic therapy into nine categories. The most common complications included phlebitis, edema, pain, burning sensation, extravasation, and bloodstream infection.

## RESUMEN

**Objetivos:**

mapear las evidencias sobre las prácticas realizadas por el equipo de enfermería en la terapia antineoplásica ambulatoria mediante el uso de catéter intravenoso periférico, e identificar las complicaciones asociadas al uso de dicho dispositivo.

**Métodos:**

se trata de una revisión de alcance siguiendo la metodología del JBI. El proceso de selección fue llevado a cabo de manera cegada por dos revisores independientes.

**Resultados:**

se recuperaron 1.287 publicaciones y, al final, se incluyeron 16 estudios. Las prácticas identificadas se relacionaron con la responsabilidad por el procedimiento; la prevención de infecciones y la bioseguridad; la selección del sitio de punción; la elección del dispositivo de punción; la preparación de la piel; la punción venosa; el mantenimiento del acceso; la cobertura y estabilización; y el retiro y descarte del dispositivo. Entre las complicaciones, se destacaron flebitis, edema, dolor, escozor, extravasación e infección del torrente sanguíneo.

**Conclusiones:**

se realizó el mapeo de las prácticas de cateterismo intravenoso periférico ambulatorio en nueve categorías, siendo las complicaciones más frecuentes la flebitis, el edema, el dolor, el escozor, la extravasación y la infección del torrente sanguíneo.

## INTRODUCTION

Systemic cancer treatment at the outpatient level improves patients’ quality of life by avoiding the disruption of family bonds caused by hospitalization, reducing hospital admissions by allowing medication infusion on-site, and enabling patients to return home upon completion of the procedure. In addition, travel to the treatment site is minimized, as patients can be treated at a facility closer to home, resulting in a significant reduction in waiting times for oncology inpatient beds^(1,2)^.

In this context, the intravenous route is widely used for Antineoplastic Therapy (AT) due to its benefits in drug absorption and serum concentration levels^(3-5)^. Administration can be performed via a central venous catheter or a PIVC, with peripheral venous infusion of antineoplastic agents being the most common approach in outpatient settings^(3,6)^.

The definition of a PIVC, according to the Infusion Nursing Society, includes devices inserted into superficial veins located just below the skin, as well as deeper veins beneath muscle tissue. These peripheral veins include those in the extremities, the external jugular vein, and scalp veins in newborns^(7)^. Due to its widespread and increasing use, the PIVC is considered one of the most common invasive procedures^(8)^.

Approximately 30% of adults experience difficulties with venipuncture during PIVC insertion, often requiring two or more attempts^(9)^. This challenging task is evident even among experienced professionals, with first-attempt failure rates ranging from 35% to 40%. Patients with difficult venous access may undergo multiple unsuccessful insertion attempts, resulting in discomfort, an increased risk of adverse events such as vascular trauma and infection, and delays in initiating AT^(6,7,9)^.

In outpatient care, the PIVC is used by the nursing team to assist individuals undergoing intravenous oncologic treatment. To ensure safe administration, nurses must have detailed knowledge of each antineoplastic drug and its potential for tissue damage. Before treatment begins, it is essential for the nurse to be attentive to individual and systemic factors that may affect AT, including a thorough assessment of the patient’s venous network and the possible harmful effects of prior intravenous treatments on the vascular endothelium^(6,10,11)^.

Many complications related to PIVC use are preventable and serve as indicators of nursing care quality. Since both the insertion and maintenance of the device are the responsibility of the nursing team, with the nurse coordinating these activities, it is crucial that this professional be prepared to implement safe practices and adopt preventive measures. The aim is to avoid adverse events that could lead to preventable complications by using the most up-to-date technologies available and following evidence-based best practices in care^(12)^.

The existing literature on AT care primarily focuses on central venous catheters for the prevention of bloodstream infections and drug extravasation. Research does not comprehensively address outpatient nursing practices, particularly regarding PIVC and the complications resulting from their use^(13-16)^. Furthermore, no scoping reviews were identified that map the current knowledge and evidence on nursing team practices in the management of peripheral catheters in outpatient settings, highlighting this study’s potential to identify gaps and guide the development of primary research.

## OBJECTIVES

To map the evidence on nursing practices in outpatient AT using PIVC and to identify complications associated with the use of this device.

## METHODS

### Ethical aspects

As this study did not involve human participants and used only publicly available literature, approval from a Research Ethics Committee was deemed unnecessary.

### Type of study

This is a scoping review guided by the JBI methodology, which also followed the PRISMA Extension for Scoping Reviews: Checklist and Explanation (PRISMA-ScR) to ensure transparency in reporting the review process^(17,18)^.

### Methodological procedure

The review protocol was registered in the Open Science Framework under DOI number: 10.17605/OSF.IO/KUM4D. The five steps followed in this scoping review were: identifying the review question, searching for relevant studies, selecting the studies, mapping the data, and presenting the results^(19)^.

Formulating the guiding question was the first step, using the PCC mnemonic: P (Population) = the nursing team; C (Concept) = peripheral intravenous catheterization practices, defined as actions related to the insertion, maintenance, removal, and disposal of the device used to administer antineoplastic medications through the peripheral venous network, and the complications resulting from PIVC use; C (Context) = AT administered in outpatient settings, classified as secondary-level care services intended for cancer treatment^(18)^. The resulting review question was: What is the available evidence on nursing practices in outpatient AT using PIVC, and what are the complications associated with the use of this device?

### Data collection and organization

The search strategy was conducted in three stages^(18)^. Initially, a limited search was carried out in the databases U.S. National Library of Medicine (PubMed), Scopus, and JBI Evidence Synthesis to identify relevant titles, indexed terms, and key descriptors derived from the PCC mnemonic. Then, the identified terms were combined with controlled vocabularies, such as Medical Subject Headings (MeSH), *Descritores em Ciências da Saúde (DeCS)*, synonyms, and keywords, using the Boolean operators ‘AND’, ‘OR’, and ‘NOT’. The search was expanded to include, in addition to the initial databases, EMBASE (Elsevier), Cochrane Library, CINAHL via EBSCOHost, *Literatura Latino-Americana e do Caribe em Ciências da Saúde (LILACS), Base de Dados em Enfermagem (BDENF)*, and Scientific Electronic Library Online (SciELO). Gray literature was explored through Open Access Theses and Dissertations (OATD) and the CAPES Catalog of Theses and Dissertations. Google Scholar was also searched to capture both gray literature and published literature in scientific journals, thereby increasing the diversity of sources.

The study selection phase consisted of screening titles and abstracts based on the inclusion criteria and the PCC mnemonic defined for this scoping review. Subsequently, the researchers conducted a thorough reading of the selected full texts, recording reasons for exclusion whenever necessary. The selection process was conducted independently and blindly by two reviewers, with disagreements resolved through consensus meetings between the reviewers. An agreement rate above 80%, as recommended in the literature, was achieved between the two reviewers, based on a pilot test conducted on a sample of 12 abstracts^(18)^.

Paid and open-access articles were included, encompassing original research, literature reviews, theses, dissertations, manuals, guidelines, and recommendations that described PIVC practices for the administration of AT in outpatient settings. These could involve the nursing team, patients, and/or other healthcare professionals, provided that nursing was explicitly mentioned in the publication. Studies focused on central venous catheters, those analyzing the administration of non-antineoplastic drugs such as antibiotics, and those not situated within the context of secondary-level outpatient care were excluded. Editorial letters, conference abstracts, and research protocols were also excluded, as they did not allow for data extraction relevant to the review question. No restrictions were applied regarding language or publication date.

The database search was conducted on August 6, 2023, and updated on February 17, 2025, following the scientific information retrieval strategy described in Chart 1. This strategy was refined after the scoping review was registered in the OSF, in accordance with the recommendations of the librarian who assisted in its development. At the end of the process, a backward search of references and citations was conducted for all selected studies, aiming to identify additional relevant publications not retrieved in the initial database searches.

**Chart 1:** Description of search strategies conducted across each source of information, February 17, 2025

Information source	Search Strategy	Publications found
PubMed	catheterization peripheral OR catheter AND antineoplastic OR antineoplastic agents AND ambulatory care AND complications NOT hospital	181
Scopus (Elsevier)	TITLE-ABS-KEY (catheterization AND peripheral AND antineoplastic OR antineoplastic AND agents AND ambulatory care OR ambulatory)	14
EMBASE (Elsevier)	(catheterization AND peripheral AND antineoplastic OR antineoplastic) AND agents AND ambulatory AND care	157
Cochrane Library	Catheterization, Peripheral OR Catheter NOT Central Venous Catheters AND Antineoplastic Agents OR Drug Therapy AND Ambulatory Care NOT Hospital in Title Abstract Keyword	275
CINAHL (EBSCO)	catheterization peripheral AND antineoplastic OR antineoplastic agents AND ambulatory care	190
LILACS	*Cateterismo Periférico OR Cateterismo venoso periférico AND antineoplásicos OR farmacoterapia OR quimioterapia AND assistência ambulatorial OR instituições de assistência ambulatorial AND ( db:(“LILACS”))*	01
BDENF	*Cateterismo Periférico OR Cateterismo Venoso Periférico AND Antineoplásicos OR Farmacoterapia OR Quimioterapia AND Assistência ambulatorial OR Instituições de Assistência Ambulatorial AND (collection_enfermeria:”BDENF” OR collection_enfermeria:”SOF-ENFERMERIA”)*	0
SciELO	Antineoplastic Agents AND Ambulatory OR Ambulatory Care NOT Hospital AND Intravenous Antineoplastic OR Chemotherapy OR Drug Therapy AND Catheter	07
Google Scholar	*Cateterismo Periférico OR Cateterismo Venoso Periférico AND Antineoplásicos OR Farmacoterapia OR Quimioterapia AND Assistência Ambulatorial OR Instituições de Assistência Ambulatorial*	450
OATD	Peripheral Catheterization AND Antineoplastic Agents OR Chemotherapy AND Ambulatory Care	01
CAPES Theses and Dissertations Catalog	Peripheral Catheterization AND Antineoplastic Agents OR Chemotherapy AND Ambulatory Care	0

In this database, the search was conducted using descriptors in English, Portuguese, and Spanish. After completing the search strategy, the information gathered from the various sources was transferred to Mendeley^®^ software (Elsevier, London, United Kingdom), and duplicates were removed. The records were then imported into Rayyan - Intelligent Systematic Review software for study selection and screening^(20)^.

The JBI recommendations were followed for data extraction^(19)^. The data from the studies selected and included in this scoping review were entered into a specific form developed by the researchers using Microsoft Excel 2022. The letter “P,” referring to the word publication, was used to classify, organize, and identify the studies. The extraction form was tested on three studies, and no adjustments were needed. The variables used for mapping the information included: authors’ last names, article publication date, country of publication, language, study design, setting/context, participants, sample size, catheter insertion and puncture practices, information on catheter maintenance, removal and disposal procedures, complications related to PIVC, and main findings (key results).

### Data analysis

The data were analyzed using descriptive statistics and presented in tables and illustrative figures^(21)^. The information was categorized to enhance understanding of nursing practices related to the use of PIVC in outpatient settings and was organized into a visual map to present the complications associated with this invasive device.

## RESULTS

**Figure 1:**
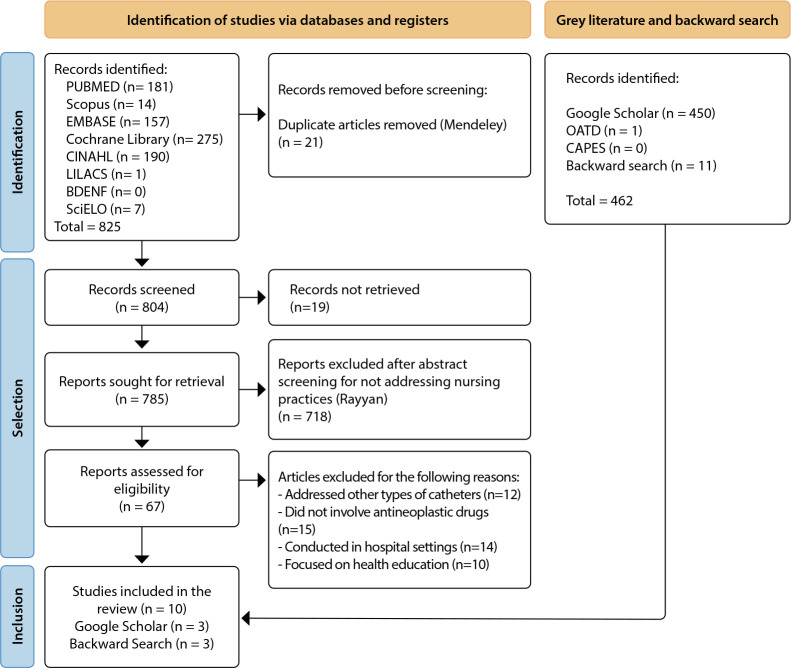
Flowchart developed based on PRISMA-ScR guidelines and adapted to include publications identified through gray literature and backward reference searching (n = 16), 2025

The search strategy applied to the databases resulted in the retrieval of 825 records, which was reduced to 804 after duplicate removal using Mendeley. After full-text screening, nine publications were identified that addressed the review question. The gray literature search yielded 451 documents, and 11 additional studies were identified through backward reference searching, totaling 1,287 publications. Sixteen studies were included in this review: ten articles identified through the database search, three publications retrieved from the gray literature (Google Scholar), and three from backward reference searching, in accordance with PRISMA-ScR guidelines (Figure 1).

No publications were identified for the periods 1980-1990, 1990-1995, and 2001-2005. In the 1996-2000 interval, two studies were found (8.7%). Between 2006-2010, two articles were published (8.7%). During the 2011-2015 period, five publications were identified (21.7%). Between 2016-2019, four studies were included (17.4%). Finally, in the period from 2020-2023, three publications were retrieved (13.0%).

Of the studies included, 56.25% were published in Brazil, while 43.75% originated from other countries, particularly the United States, Portugal, Belgium, and the United Kingdom. Descriptive studies represented approximately 47% of the methodological designs, followed by integrative review studies (17.64%) and exploratory studies (17.64%). In the gray literature, three guidelines were identified (18.75%), two of which were international and one national.

Chart 2 provides details on the selected publications, including authors, title, journal of publication, sample size, and participants.

**Chart 2:** Characterization of the publications included in the scoping review, 2025 (N = 16)

ID^*^	Authors	Title	Journal	Sample	Participants
P1†	Nóbrega et al.^(22)^	Custo de procedimentos relacionados ao tratamento quimioterápico ambulatorial de mulheres portadoras de câncer de mama	Rev Esc Enferm USP	60 procedures observed in five stages	Nurses
P2†	Selwood et al.^(23)^	*Vascular access for daunorubicin during childhood acute lymphoblastic leukaemia induction treatment: a UKCCSG supportive care group and MRC childhood leukaemia working party survey*	Eur J Oncol Nurs	22 pediatric oncology centers	Pediatric hematologists and healthcare professionals
P3†	Silva et al.^(24)^	A visão dos enfermeiros acerca dos acessos venosos para administração da quimioterapia	Rev enferm UFPE on line	10 participants	Nurses
P4†	Dias et al.^(25)^	Padrões de cuidados em prevenção e tratamento de extravasamento de antineoplásicos baseado em evidências clínicas	Rev Enf Atual	30 participants	Studies addressing antineoplastic extravasation and infiltration
P5†	Fernandes et al.^(26)^	Mulheres mastectomizadas em vigência de quimioterapia adjuvante: assistência do enfermeiro	Perspectivas Online: Biológicas & Saúde	30 participants	Mastectomized women aged 30-45 years
P6†	Passos^(27)^	Validação de um sistema de classificação de pacientes para a prestação de cuidados de enfermagem em ambulatório de oncologia	Repositório Iscte Instituto Universitário de Lisboa	7 participants	Nurses
P7†	Freitas^(28)^	Estratégias para administração segura de antineoplásicos	Repositório Institucional Universidade Estadual Paulista	30 participants	Nurses
P8†	Nakamura et al.^(29)^	Avaliação dos cuidados de enfermagem em uma unidade de quimioterapia	Revista HCPA. Porto Alegre	144 procedures performed by rofessionals	Nurses
P9†	Costa^(30)^	Assistência da equipe de enfermagem a pacientes oncológicos em uso de cateteres: uma revisão integrativa	Repositório Digital - URI Erechim	7 articles	Scientific publications on nursing care for cancer patients using catheters
P10†	Rodrigues^(31)^	Fatores de risco para trauma vascular durante a quimioterapia antineoplásica: contribuições do emprego do risco relativo^*^	Acta Paul. de Enferm	30 participants	Patients undergoing chemotherapy for breast cancer
P11†	Oslon et al.^(32)^	*Intravenous therapy needle choices in ambulatory cancer patients*	Clin Nurs Res	100 participants	Patients receiving antineoplastic treatment
P12†	Shotkin et al.^(33)^	*Use of an indwelling peripheral catheter for 3-5 day chemotherapy administration in the outpatient setting.*	J Intraven Nurs	89 participants	Patients receiving 3-5 day antineoplastic treatment
P13†	Reis et al.^(34)^	Efeitos adversos identificados em local de infusão intravenosa periférica por drogas quimioterápicas	Ciencia y Enfermería XIV	15 participants	Patients receiving peripheral antineoplastic treatment
P14†	Perez Fidalgo et al.^(35)^	*Management of chemotherapy extravasation: ESMO-EONS Clinical Practice Guidelines*	European Journal of Oncology Nursing	Not applicable	Guideline
P15†	Anvisa^**(36)^	Práticas seguras para a prevenção de incidentes envolvendo cateter intravenoso periférico em serviços de saúde	Agência Nacional de Vigilância Sanitária	Not applicable	Technical note
P16†	Gorski, LA. et al.^(37)^	*Infusion therapy standards of practice*	Journal of Infusion Nursing	Not applicable	Guideline

* ID - Identification; †P - Publication;

** Brazilian Health Regulatory Agency (Agência Nacional de Vigilância Sanitária).

* *PIVC - Peripheral Intravenous Catheter.*

The selected publications presented various nursing practices related to the use of PIVC for AT in outpatient settings^(22-37)^. These practices were organized into nine categories: responsibility for the procedure, infection prevention and biosafety, selection of the puncture site, selection of the puncture device, skin preparation, venous puncture, access maintenance, catheter dressing and stabilization, and device removal and disposal, as shown in Chart 3.

**Chart 3:** Description of nursing practices related to outpatient Antineoplastic Therapy using peripheral intravenous catheterization, 2025

Category	Nursing Practices
Responsibility for the procedure^(22-24)^	- To be performed by nurses or nursing technicians.
Infection prevention and biosafety^(31,36)^	- Perform hand hygiene before and after the procedure; - Use personal protective equipment.
Selection of the puncture site^(31,34-37)^	- Identify a limb with no restrictions, not affected by lymphadenectomy, arteriovenous fistula, edema, hemiplegia, or hemiparesis; - Avoid lower limbs, favor upper limbs; - Select a large, palpable, non-tortuous vein ideally located in areas that protect joints; avoid regions near tendons, nerves, and those that may suffer more anatomical damage if extravasation occurs; - Follow the preferential order for puncture: forearm, back of the hand, wrist, and antecubital fossa.
Selection of the puncture device^(23,24,27-36)^	- Choose winged or non-winged device for puncture; - Select a device compatible with the chosen vein, using the smallest possible gauge that supports therapy infusion.
Skin preparation^(23,25,27,33,34-37)^	- Rub the skin with an alcohol-based solution (0.5% chlorhexidine gluconate, 10% alcoholic PVP-I, or 70% alcohol), using the proper technique for each solution; - Allow spontaneous drying; - Do not touch the puncture site after skin antisepsis.
Venous puncture^(24,25,31,34-37)^	- Apply a tourniquet to the limb for less than two minutes; - Use a 15º angle for device insertion; - Limit puncture attempts to two per professional and five per patient.
Access maintenance^(25,26,29,31,34-37)^	- Check for blood return and flow immediately after puncture and routinely before, during, and after drug administration; - Supervise vesicant drug infusion; - Flush the vein between drugs and after infusion with saline or glycosaline solution; - Continuously monitor the catheter insertion site for early signs of complications and the need for device replacement.
Dressing and stabilization^(23,27,31,32,34-37)^	- Use a sterile dressing that allows ostium visualization, ensuring safety and stabilization of the device.
Removal and disposal^(23,29,33,35-37)^	- Immediately discard the needle from the non-winged device and the winged device into a sharps container; - Immediately discard the flexible catheter, without disconnecting it from the infusion circuit, into chemical waste.

* *PVP-I - Povidone-iodine.*

**Figure 2:**
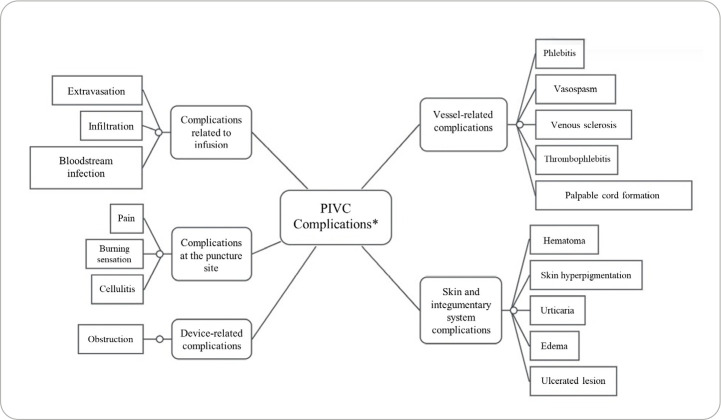
Map of complications related to Peripheral Intravenous Catheter use for Antineoplastic Therapy in outpatient settings, 2025

Figure 2 presents the map of complications related to PIVC use. Among the complications associated with the vein, the following were highlighted: phlebitis^(22,29,31,32)^, venous sclerosis^(22,34)^, vasospasm^(31)^, thrombophlebitis^(31)^, and formation of a palpable cord^(32)^. Regarding the skin and integumentary system, the identified complications included edema^(25,31)^, hematoma^(28,34)^, ulcerated lesion^(22,31)^, skin hyperpigmentation^(28)^, and urticaria^(22)^. Complications identified at the puncture site included pain^(22,24,25,28,30,32)^, burning sensation^(24,27,34)^, and cellulitis^(31)^. Complications associated with infusion included extravasation^(23,24,32,34,35)^, infiltration^(29,31)^, and bloodstream infection^(29-32)^. Obstruction was the only complication resulting directly from the device itself^(27)^.

## DISCUSSION

This study mapped the available evidence in the literature on nursing practices related to AT using PIVC in outpatient settings and the complications associated with the use of this device, presenting a categorized synthesis of the findings.

The predominance of descriptive studies among the publications reflects a limitation in the production of more robust research, such as clinical trials and longitudinal studies. Although these publications provide a general overview of the practices, they do not explore in depth the effectiveness of the recommended interventions, which could help to consolidate stronger scientific evidence for the prevention and management of PIVC-related complications.

The analysis of the findings did not reveal significant changes in nursing practices or complications related to PIVC use for outpatient AT in the post-COVID-19 period, as only three publications were retrieved between 2020 and 2023, with no direct comparative approaches. Technical Note GVIMS/GGTES/ANVISA No. 04/2022 reinforces guidelines for the safe use of PIVC, while the *Infusion Therapy Standards of Practice*, published in 2021, establishes international standards for infusion therapy. An integrative review also synthesizes existing evidence; however, none of these documents assess specific changes before and after the pandemic^(30,36,37)^. Nonetheless, it is plausible to consider that this period reinforced biosafety measures, the use of personal protective equipment, and the need for professional training-suggesting the importance of future studies to evaluate possible impacts on complication rates, protocol adherence, and the qualification of nursing professionals.

Two studies addressed the responsibility for performing the procedure. The study by P2 assigned this function to both nurses and nursing technicians^(22,23)^. However, the study by P3 stated that only nurses were responsible for venipuncture^(24)^. At the national level, no specific resolution from the Federal Nursing Council (COFEN in Portuguese) was identified regarding responsibility for peripheral venous puncture procedures. Nevertheless, an opinion issued by the Regional Nursing Council of the State of São Paulo (COREN-SP in Portuguese) indicates that the procedure may be performed by the nursing team, with the nurse being responsible for assessing the patient’s venous condition prior to the procedure. The opinion further recommends that, in cases of difficult venous access, the procedure should preferably be performed by the nurse^(38)^.

Regarding PIVC management in outpatient settings, the studies highlighted several nursing practices. Hand hygiene was described as the main biosafety and infection prevention strategy related to healthcare-associated infections. Although adherence to proper technique was not assessed, study P10 emphasizes this preventive measure as a care practice for peripheral catheters, which should be routinely performed and monitored by the nursing team^(31)^. The procedure must be carried out whenever the device is handled-before, during, and/or after use. The appropriate technique may vary in duration depending on the method used: 40 to 60 seconds when using water and liquid soap, and 20 to 30 seconds when using an alcohol-based solution^(39)^.

Regarding the choice of puncture site, actions focused on the careful assessment of the limb were identified, aiming to select an appropriate vein. Studies P10 and P13 emphasize the importance of avoiding limbs with restrictions, such as those affected by lymphadenectomy, arteriovenous fistula, edema, hemiplegia, or hemiparesis, and consistently favoring the upper limbs^(31,34-37)^. These findings are consistent with a Brazilian study, which reported no punctures in areas with infected, broken, or inflamed skin, or near bony structures and flexion regions, and recommended catheterization in the upper limbs to prevent complications^(40)^.

After identifying the appropriate limb for puncture, study P4 highlighted the selection of a large, palpable, and non-tortuous vein as the main criteria^(24,34-37)^. Study P10 supports this approach, recommending veins located in areas that protect joints, while avoiding regions close to tendons and nerves, and minimizing the risk of anatomical damage in the event of extravasation^(31)^. Although there is no consensus on the ideal site, study P13 suggests the use of veins in the forearm and the back of the hand^(34)^. However, certain areas should be avoided, as the use of poorly visible, tortuous, and non-palpable veins was observed^(30)^, as well as punctures in the lower limbs of adult patients^(34)^.

In this context, the most current guideline recommendations align with the practices observed regarding the choice of limb and vein to be punctured. The veins recommended for PIVC insertion in adults include those located on the dorsal and ventral surfaces of the forearms, such as the cephalic, basilic, and median antebrachial veins, the elbow, and the dorsum of the hand-provided the upper limbs are free of conditions that may compromise the safety of the prescribed infusion therapy^(36,37)^.

Regarding the choice of the device to be inserted into the vein, three studies mentioned the use of winged and non-winged catheters without specifying the gauge^(23,24,29)^. Study P13 used winged catheters with gauges 21, 23, and 25, and non-winged catheters with a 24-gauge^(34)^. Other researchers specified the use of Vialon catheters in gauges 20, 22, and 24^(33)^. In this regard, one point of concern is the need to avoid the use of plastic winged and non-winged catheters^(32)^, as the literature recommends using non-winged catheters made of inert, non-thrombogenic, flexible, radiopaque or transparent material, with the smallest possible gauge^(28)^.

Study P4 highlights the practice of selecting a puncture device compatible with the chosen vein^(25)^, and other researchers emphasize that, beyond compatibility, there is a concern with choosing the smallest possible gauge that can support the infusion therapy^(30)^. In this regard, to improve vein visualization, two studies reported the use of a tourniquet to promote vein engorgement^(27,31)^. Supporting this, study P13 specifies that a tourniquet is used and that its application time should be less than two minutes-an aspect to be evaluated by the nursing team to avoid hemolysis and increased intravascular pressure^(34)^.

Several studies emphasize the importance of selecting the PIVC based on the intended purpose, duration of therapy, fluid viscosity, and venous access conditions. Smaller gauge catheters are associated with a lower risk of mechanical and chemical phlebitis. In such cases, the use of flexible catheters is recommended, while steel cannulas should be avoided due to their higher risk of vessel perforation and medication extravasation^(35-37,41)^.

Regarding skin preparation for puncture, studies P2, P6, and P13 report the use of 70% alcohol-based antiseptics^(23,27,34)^, with a recommendation to allow the solution to dry spontaneously before initiating the puncture^(27)^. Study P11 describes antisepsis using 10% povidone-iodine followed by 70% alcohol, which is then dried with sterile gauze^(32)^. In addition to proper skin preparation, study P4 highlights the practice of avoiding palpation of the puncture site after antisepsis and during the puncture procedure^(25)^.

Skin preparation is indicated for the prevention of bacterial phlebitis and infections related to device use. The antiseptic solutions identified in practice were consistent with those recommended in the literature; however, it is important to note that if visible dirt is present, the area should be cleaned with soap and water or 2% chlorhexidine before proceeding with alcohol-based antisepsis. Regardless of the antiseptic used, spontaneous drying must be allowed before performing the puncture, and the site should not be touched again^(36,37)^.

One study describes the insertion angle of the catheter device during puncture, recommending a 15º angle between the bevel of the catheter needle and the patient’s skin^(23)^. In cases of unsuccessful attempts, study P6 was the only one to address a limit on puncture attempts, recommending two attempts per professional and a total of five per patient^(27)^. A different perspective is presented in study P3, which states that there is no defined limit on attempts to obtain PIVC, but emphasizes that the correct angle and technique must be followed by the nursing team^(24)^. Nevertheless, it is recommended to use a new catheter for each puncture attempt, as reuse may result in loss of lubrication, obstruction, and increased risk of complications^(36,40)^.

Among the studies included in this review, there was no evidence of the use of visualization technologies for venous puncture. Performing blind attempts without the aid of such technologies may increase the number of attempts required to achieve successful access, potentially leading to depletion of the peripheral venous network^(42)^. Current literature recommends the use of techniques such as ultrasound, visible light devices, and near-infrared light in patients with difficult venous access or following unsuccessful puncture attempts, as these technologies have been associated with higher success rates on the first PIVC insertion attempt^(36,37,43)^.

Immediately after puncture, study P4 describes the practice of checking for blood return and flow only before initiating the infusion of AT^(25)^. In addition to confirming access patency prior to administering the antineoplastic agent, other researchers include two additional testing points: during the infusion and at the end of therapy^(29)^. Another publication mentions checking for flow and blood return during the infusion^(34)^, indicating that this is a common practice across the three studies conducted at different times.

Regarding PIVC dressing, study P2 mentions the use of sterile transparent materials or microporous tape^(23)^, while P10 refers only to transparent dressing, without specifying sterility^(31)^. It is worth noting that study P11 identifies different types of dressings depending on the catheter type: flexible catheters are covered with occlusive dressings, and winged catheters with nylon tape^(32)^. From another perspective, study P6 - more recently published than the others - describes the practice of selecting dressings that provide stabilization, are sterile, allow ostium visualization, and ensure safety^(27)^.

Some findings align with a study indicating that sterile dressings are most commonly used for peripheral venous catheters^(44)^. However, the use of non-sterile dressings was reported in two studies, which is not recommended due to the risk of microbial contamination. For covering peripheral catheters, the ideal practice is to use a sterile dressing with gauze and adhesive tape or a transparent membrane^(36,37)^.

Studies P1 and P2 cite surveillance during the administration of antineoplastic drugs, emphasizing the importance of continuous monitoring of vesicant drugs administered via PIVC^(22,23)^. For this reason, maintenance practices during infusion focus on preventing complications and recognizing them early if they occur.

Regarding vein irrigation, studies P2 and P4 describe flushing the venous access with 0.9% saline solution between medications and at the end of AT^(23,25)^. Although there was no consensus on the volume to be used, study P2 suggests using 50 mL of the solution for this practice^(23)^. Exploring other suggested solutions, one study mentions using either 0.9% saline or distilled water between drugs and at the end of infusion^(29)^, while another publication (P13) adds glycosaline solution before and after the antineoplastic agents, though neither specifies the volume used^(34)^.

As PIVC is used in outpatient settings, researchers (P5 and P10) emphasize the importance of continuous monitoring of the catheter insertion site to detect early signs of complications. In this context, they highlight the need to replace the device in cases of phlogistic signs or other adverse events^(26,31)^.

Regarding the removal and disposal of the PIVC, study P2 reports that for winged devices, disposal occurs immediately after the infusion ends, using a sharps container designated for chemical waste. In contrast, for non-winged catheters, the needle is discarded immediately after puncture into a sharps container designated for infectious waste. At the end of the infusion, the entire circuit is discarded into a plastic bag intended for Group B waste, classified as chemical risk^(23)^. Study P8 discourages certain practices observed in healthcare settings, illustrating that disposal was not performed immediately after catheter removal, with the puncture device left hanging from the IV pole near the patient and other professionals^(29)^.

Regarding the catheter replacement interval, only study P12 reported the practice of maintaining the PIVC for three to five days in patients who returned to the outpatient clinic on subsequent days for infusion. Thus, for device maintenance, the daily administration of a 2 mL heparin lock is suggested^(33)^. Current guidelines are consistent with these practices, reinforcing the safety and effectiveness of device maintenance^(36)^.

Some important aspects of general care practices related to PIVC were identified in the study conducted by P10. It is recommended to avoid excessive accessories connected to the infusion circuit, as this may overload the intravenous device. Additionally, the infusion of vesicant drugs via continuous infusion pumps through peripheral lines should be avoided. An important recommendation is to avoid prolonged infusion (lasting more than 30 minutes) of vesicant drugs through the device and not to use veins that were punctured more than 24 hours earlier^(31)^.

Regarding complications associated with PIVC use, the most frequently reported were phlebitis, infiltration, and extravasation. Catheter occlusion or displacement and pain were also commonly reported. Finally, more serious adverse outcomes included local infection, catheter-related bloodstream infection, and extravasation-particularly involving anthracyclines, which can result in more severe tissue injury^(22,24-26,28,30,32)^.

Based on an analysis of the studies reporting complications, it is emphasized that the reporting of these adverse events associated with outpatient AT via PIVC is essential for analyzing incidents and adverse outcomes. This practice enables proper management and mitigation of their occurrence by implementing evidence-based practices and eliminating outdated ones considered risky to procedural safety^(28)^. A systematic review conducted in 2023 supports and reinforces that the notification of complications is critical for managing healthcare-related risks that compromise patient safety^(45)^.

### Study limitations

Regarding the study’s limitations, the restriction of descriptors focused on the outpatient setting should be considered, as it may have led to the exclusion of relevant studies during the search strategy.

### Contributions to the Field

This study may contribute to the body of knowledge on nursing care practices involving PIVC for outpatient AT, as well as its associated complications. Although scoping reviews are not intended for evidence implementation, it is noteworthy that the findings may support patient care strategies, particularly for those who rely on secondary-level care to meet needs related to cancer treatment.

## CONCLUSIONS

This study enabled the mapping, in the literature, of peripheral intravenous catheterization practices for the administration of outpatient AT, as well as complications associated with the use of the device. Nursing practices were identified that may guide actions within healthcare services and support discussion of the findings disseminated in the literature. These practices include responsibility for the procedure, infection prevention and biosafety, selection of the puncture site, choice of puncture device, skin preparation, venous puncture, access maintenance, catheter dressing and stabilization, and device removal and disposal.

In addition to these practices, complications such as phlebitis, edema, pain, burning sensation, extravasation, and bloodstream infection were highlighted. This research presented the state of the art using the scoping review methodology and will serve as a guide for patient care, helping address demands related to cancer treatment in secondary healthcare settings.

## Data Availability

The research data are available within the article.
