# Supplementary material for: Nursing practices and complications associated with peripheral catheterization for outpatient antineoplastic therapy: scoping review
Source: Rev Bras Enferm. 2025 Dec 8;78(Suppl 4):e20240528. doi: 10.1590/0034-7167-2024-0528 (PMC12700578; doi:10.1590/0034-7167-2024-0528)
Supplement: Translation

# Translation: Práticas de enfermagem e complicações associadas ao cateterismo periférico para terapia antineoplásica ambulatorial: revisão de escopo

**DOI:** 10.1590/0034-7167-2024-0528pt

**Authors:** Vanessa Albuquerque Alvim de Paula, Vilanice Alves de Araújo Püschel, Kelli Borges dos Santos, Fábio da Costa Carbogim, André Luiz Silva Alvim

**Affiliations:** I Universidade Federal de Juiz de Fora. Juiz de Fora, Minas Gerais, Brasil; II Universidade de São Paulo. São Paulo, São Paulo, Brasil; III Centro Brasileiro para o Cuidado à Saúde em Evidências: Centro de Excelência do JBI (JBI Brasil). São Paulo, São Paulo, Brasil

**Keywords:** Cateterismo Periférico, Antineoplásicos, Assistência Ambulatorial, Enfermagem, Complicações.

## Abstract

**Objetivos:**

mapear as evidências sobre as práticas realizadas pela equipe de enfermagem na terapia antineoplásica ambulatorial com o uso de cateter intravenoso periférico e identificar as complicações associadas ao uso desse dispositivo.

**Métodos:**

trata-se de uma revisão de escopo seguindo a metodologia do JBI. O processo de seleção foi conduzido de forma cegada por dois revisores independentes.

**Resultados:**

foram recuperadas 1.287 publicações e, ao final, incluídos 16 estudos. As práticas foram relacionadas à responsabilidade pelo procedimento; prevenção de infecção e biossegurança; seleção do sítio de punção; escolha do dispositivo de punção; preparo da pele; punção venosa; manutenção do acesso; cobertura e estabilização; e retirada e descarte do dispositivo. Entre as complicações, destacaram-se flebite, edema, dor, ardência, extravasamento e infecção da corrente sanguínea.

**Conclusões:**

foi realizado o mapeamento das práticas de cateterismo intravenoso periférico ambulatorial em nove categorias, sendo as complicações mais frequentes flebite, edema, dor, ardência, extravasamento e infecção da corrente sanguínea.

## INTRODUÇÃO

O tratamento sistêmico do câncer em nível ambulatorial possibilita a melhora da qualidade de vida do paciente, pois evita a quebra do vínculo familiar decorrente da hospitalização, reduz internações hospitalares ao permitir a infusão de medicamentos no local e o retorno para o domicílio após término do procedimento. Além disso, há diminuição do deslocamento até o local de tratamento, uma vez que o paciente pode se inserir em um ambulatório próximo à sua residência, resultando em uma significativa redução do tempo de espera por vagas em enfermarias oncológicas^(1,2)^.

Nesse contexto, a via endovenosa é amplamente utilizada para a Terapia Antineoplásica (TA), pelos benefícios na absorção e nos níveis séricos da droga^(3-5)^. A administração pode ser realizada por meio de cateter venoso central ou cateter intravenoso periférico(CIVP), sendo a infusão de antineoplásicos através da rede venosa periférica a abordagem mais comum em ambulatórios^(3,6)^.

A definição de CIVP pela *Infusion Nursing Society* engloba dispositivos inseridos em veias superficiais, localizadas logo abaixo da pele, e em veias mais profundas, sob o tecido muscular. Essas veias periféricas incluem as extremidades, a veia jugular externa e as veias do couro cabeludo em recém-nascidos^(7)^. Devido à sua ampla utilização e ao aumento progressivo de seu uso, o CIVP é considerado um dos procedimentos invasivos mais populares^(8)^.

Aproximadamente 30% dos adultos enfrentam dificuldades para punção venosa na inserção do CIVP, necessitando de duas ou mais tentativas^(9)^. Essa tarefa desafiadora é evidente mesmo para profissionais experientes, com taxas de falha na primeira tentativa entre 35% e 40%. Pacientes com acesso venoso difícil sofrem sucessivas tentativas malsucedidas de inserção do CIVP, acarretando desconforto, aumento do risco de eventos adversos, como trauma vascular e infecção, além de atraso no início da TA^(6,7,9)^.

No contexto ambulatorial, o CIVP é usado pela equipe de enfermagem nos cuidados de pessoas em terapêutica oncológica endovenosa. Para assegurar a administração segura, o enfermeiro deve ter conhecimento detalhado de cada antineoplásico e seus possíveis danos teciduais. Antes do início do tratamento, é essencial que esteja atento aos fatores individuais e sistêmicos que podem influenciar a TA, incluindo uma avaliação criteriosa da condição da rede venosa do paciente e possíveis efeitos prejudiciais de tratamentos endovenosos prévios sobre o endotélio vascular^(6,10,11)^.

Muitas complicações relacionadas ao CIVP são evitáveis e servem como indicadores de qualidade na assistência de enfermagem. Dado que a inserção e manutenção do dispositivo são responsabilidades da equipe de enfermagem, com o enfermeiro coordenando esses cuidados, torna-se crucial que este profissional esteja preparado para estabelecer práticas seguras e adotar medidas preventivas. Isso visa evitar eventos adversos que possam predispor a complicações evitáveis, utilizando as tecnologias mais recentes disponíveis e seguindo as melhores práticas de cuidado recomendadas^(12)^.

A literatura existente sobre os cuidados na TA destaca predominantemente os cateteres venosos centrais para prevenção de infecções da corrente sanguínea e extravasamento de drogas. As pesquisas não abordam de maneira abrangente as práticas da equipe de enfermagem em ambulatórios, especialmente em relação ao CIVP e às complicações decorrentes do uso desse dispositivo^(13-16)^. Somado a isso, não foram identificadas revisões de escopo que mapeassem o conhecimento e as evidências existentes sobre as práticas da equipe de enfermagem no manejo do cateter periférico na atenção ambulatorial, destacando o potencial desta pesquisa para identificar lacunas e orientar o desenvolvimento de estudos primários.

## OBJETIVOS

Mapear as evidências sobre as práticas realizadas pela equipe de enfermagem na terapia antineoplásica ambulatorial, com o uso de CIVP, e identificar as complicações associadas ao uso desse dispositivo.

## MÉTODOS

### Aspectos éticos

Por se tratar de uma pesquisa que não envolveu seres humanos e utilizou apenas publicações de domínio público, disponíveis na literatura, a obtenção de parecer do Comitê de Ética em Pesquisa foi dispensável.

### Tipo de estudo

Trata-se de uma revisão de escopo guiada pela metodologia do Joanna Briggs Institute (JBI), que utilizou, para orientar a transparência no relato da revisão, as diretrizes do *PRISMA Extension for Scoping Reviews: Checklist and Explanation (PRISMA-ScR)* ^(17,18)^.

### Procedimento metodológico

O protocolo da revisão foi registrado na Open Science Framework com o número DOI: 10.17605/OSF.IO/KUM4D. Os cinco passos adotados nesta revisão de escopo foram: identificação da pergunta de revisão, levantamento de estudos relevantes, seleção dos estudos, mapeamento dos estudos e apresentação dos resultados^(19)^.

A formulação da pergunta norteadora constitui-se como a primeira etapa, utilizando o mnemônico PCC, sendo P (Participante) = a equipe de enfermagem; C (Conceito) = as práticas de cateterismo intravenoso periférico, definidas como as ações voltadas à inserção, manutenção, retirada e descarte do dispositivo utilizado para administração de medicamentos antineoplásicos na rede venosa periférica, e as complicações decorrentes do uso do CIVP; C (Contexto) = a TA administrada em ambulatórios, sendo classificada como serviços de atenção secundária destinados ao tratamento oncológico^(18)^. A pergunta de revisão resultante foi: *Quais as evidências disponíveis sobre as práticas realizadas pela equipe de enfermagem em nível ambulatorial na TA utilizando CIVP e as complicações associadas ao uso do dispositivo?*

### Coleta e organização dos dados

A estratégia de busca foi realizada em três etapas^(18)^. Inicialmente, uma busca limitada foi conduzida nas bases de dados U*.S. National Library of Medicine* (PubMed), Scopus e *JBI Evidence Synthesis*, com o objetivo de identificar títulos, termos indexados e descritores principais derivados do mnemônico PCC. Em seguida, os termos identificados foram combinados com vocabulários controlados, como *Medical Subject Headings* (MeSH), Descritores em Ciências da Saúde (DeCS), sinônimos e palavras-chave, utilizando os operadores booleanos *‘AND’, ‘OR’* e *‘NOT’*. A pesquisa eletrônica foi ampliada para incluir, além das duas primeiras, as bases de dados EMBASE (Elsevier), *Cochrane Library*, CINAHL via EBSCOHost, Literatura Latino-Americana e do Caribe em Ciências da Saúde (LILACS), Base de Dados em Enfermagem (BDENF) e *Scientific Electronic Library Online* (SciELO). A literatura cinza foi consultada por meio do *Open Access Theses and Dissertations* (OATD) e do Catálogo de Teses e Dissertações da Coordenação de Aperfeiçoamento de Pessoal de Nível Superior (CAPES). A pesquisa no Google Acadêmico abrangeu tanto literatura cinza quanto literatura publicada em periódicos científicos, ampliando a diversidade das fontes consultadas.

A etapa de seleção dos estudos consistiu na avaliação dos títulos e resumos, baseando-se nos critérios de inclusão e no mnemônico PCC definido nesta revisão de escopo. Posteriormente, os pesquisadores realizaram uma leitura minuciosa dos textos selecionados, registrando, em todos os momentos necessários, os motivos de exclusão. O processo de seleção foi conduzido de forma cega por dois revisores independentes, com as divergências resolvidas por meio de uma reunião entre os próprios pesquisadores. O percentual de concordância, superior a 80%, conforme recomendado pela literatura, foi alcançado entre os dois revisores, considerando que o estudo foi testado em uma amostra de 12 títulos de resumos^(18)^.

Foram incluídos artigos pagos e de acesso gratuito relacionados a pesquisas originais, revisões de literatura, teses, dissertações, manuais, diretrizes e *guidelines* que descreveram as práticas de CIVP para administração da TA em ambulatórios, seja com a equipe de enfermagem ou com os pacientes e/ou outros profissionais de saúde, desde que fosse explícita a menção à enfermagem na publicação. Foram excluídos estudos voltados aos cateteres venosos centrais, que analisavam a administração de outras drogas não antineoplásicas, como antibióticos, e que não tinham como contexto a atenção secundária (ambulatório), além de cartas ao editor, resumos em anais de eventos e protocolos de pesquisa, por não possibilitarem a extração de dados para responder à pergunta de revisão. Não houve restrição de idioma e tempo para a busca.

A busca nas bases de dados foi realizada em 6 de agosto de 2023 e atualizada em 17 de fevereiro de 2025, seguindo a estratégia de recuperação de informação científica descrita no Quadro 1. Essa estratégia foi ajustada após o registro da revisão de escopo na OSF, em conformidade com as sugestões da bibliotecária que auxiliou em sua elaboração. Ao término, foi realizada uma busca reversa nas referências e citações dos estudos selecionados para a extração de dados em todas as publicações selecionadas para este estudo, buscando captar estudos não contemplados nas bases de dados selecionadas.

**Quadro 1:** Descrição das estratégias de busca realizadas em cada fonte de informação, 17 de fevereiro de 2025

Fonte de informação	Busca realizada	Publicações encontradas
PubMed	*catheterization peripheral OR catheter AND antineoplastic OR antineoplastic agents AND ambulatory care AND complications NOT hospital*	181
Scopus (Elsevier)	*TITLE-ABS-KEY (catheterization, AND peripheral AND antineoplastic OR antineoplastic AND agents AND ambulatory care OR ambulatory)*	14
EMBASE (Elsevier)	*(catheterization AND peripheral AND antineoplastic OR antineoplastic) AND agents AND ambulatory AND care*	157
Cochrane Library	*Catheterization, Peripheral OR Catheter NOT Central Venous Catheters AND Antineoplastic Agents OR Drug Therapy AND Ambulatory Care NOT Hospital in Title Abstract Keyword*	275
CINAHL (EBSCO)	*catheterization peripheral AND antineoplastic OR antineoplastic agents AND ambulatory care*	190
LILACS^*^	Cateterismo Periférico *OR* Cateterismo venoso periférico *AND* antineoplásicos *OR* farmacoterapia *OR* quimioterapia *AND* assistência ambulatorial *OR* instituições de assistência ambulatorial *AND* ( db:(“LILACS”))	01
BDENF	Cateterismo Periférico *OR* Cateterismo Venoso Periférico *AND* Antineoplásicos *OR* Farmacoterapia *OR* Quimioterapia *AND* Assistência ambulatorial *OR* Instituições de Assistência Ambulatorial *AND* (*collection_enfermeria:”BDENF” OR collection_enfermeria:”SOF-ENFERMERIA*”)	0
SciELO	*Antineoplastic Agents AND Ambulatory OR Ambulatory Care NOT Hospital AND Intravenous Antineoplastic OR Chemotherapy OR Drug Therapy AND Catheter*	07
Google Acadêmico	Cateterismo Periférico *OR* Cateterismo Venoso Periférico *AND* Antineoplásicos *OR* Farmacoterapia *OR* Quimioterapia *AND* Assistência Ambulatorial *OR* Instituições de Assistência Ambulatorial	450
OATD	Cateterismo Periférico *AND* Antineoplásicos *OR* Quimioterapia *AND* Assistência Ambulatorial	01
Catálogo de Teses e Dissertações (CAPES)	Cateterismo Periférico *AND* Antineoplásicos *OR* Quimioterapia *AND* Assistência Ambulatorial	0

* *Nesta base de dados, a busca foi realizada utilizando os descritores em inglês, português e espanhol.*

Após a realização da estratégia de busca, as informações levantadas nas diferentes fontes foram transferidas para o *software* Mendeley^®^ (Elsevier, London, United Kingdom) e as duplicadas foram removidas. Em seguida, os registros foram importados para o software Rayyan - *Intelligent Systematic Review*, para seleção e exploração dos estudos^(20)^.

As recomendações do JBI foram empregadas para a extração de dados^(19)^. As informações dos estudos selecionados e incluídos nesta revisão de escopo foram inseridas em um formulário específico, desenvolvido pelos pesquisadores no Microsoft Excel 2022, no qual a letra “P”, referindo-se à palavra *publicação*, foi utilizada para classificação, organização e identificação dos estudos. O formulário de extração foi testado em três estudos, e nenhum ajuste foi necessário. As variáveis para o mapeamento das informações foram: sobrenome dos autores, data de publicação do artigo, país de publicação, idioma, desenho do estudo, ambiente/contexto, participantes, tamanho da amostra, práticas de punção e inserção do cateter, descrição sobre a manutenção do dispositivo, informações sobre a remoção e descarte, complicações relacionadas ao CIVP e principais destaques (resultados alcançados).

### Análise dos dados

Os dados foram analisados utilizando estatística descritiva e apresentados por meio de quadros e figuras explicativas^(21)^. As informações foram categorizadas para aprimorar a compreensão das práticas de enfermagem relacionadas ao uso do CIVP em ambientes ambulatoriais e organizadas em um mapa visual para a apresentação das complicações associadas a esse dispositivo invasivo.

## RESULTADOS

A estratégia de busca nas bases de dados resultou na recuperação de 825 registros, número reduzido para 804 após a eliminação de duplicatas no software Mendeley. Após a análise dos textos completos, identificaram-se nove publicações que responderam à pergunta de revisão. A literatura cinza explorada resultou em 451 documentos, e 11 foram identificados por meio da busca reversa. Ao todo, foram 1.287 publicações, sendo incluídos nesta revisão 16 estudos, estratificados em: 10 artigos encontrados na estratégia de busca, três publicações identificadas na literatura cinza (Google Acadêmico) e três na busca reversa, seguindo as recomendações do PRISMA-ScR (Figura 1).

**Figura 1:** Fluxograma elaborado com base nas recomendações do PRISMA-ScR e adaptado para acrescentar as publicações identificadas na literatura cinza e na busca reversa (n = 16), 2025

Não foram identificadas publicações nos períodos de 1980-1990, 1990-1995 e 2001-2005. No intervalo de 1996-2000, foram encontrados dois estudos (8,7%). Entre 2006-2010, dois artigos foram publicados (8,7%). No período de 2011-2015, houve cinco publicações (21,7%). Entre 2016-2019, foram identificados quatro estudos (17,4%). Por fim, no período de 2020-2023, três publicações foram recuperadas (13,0%).

Dos estudos incluídos, 56,25% foram publicados no Brasil, enquanto 43,75% eram oriundos de outros países, em especial os Estados Unidos, Portugal, Bélgica e Reino Unido. Os estudos descritivos compreendem o delineamento metodológico de aproximadamente 47% do total, seguidos por estudos de revisão integrativa (17,64%) e exploratórios (17,64%). Na literatura cinza, foram identificadas três diretrizes (18,75%), sendo duas internacionais e uma nacional.

O Quadro 2 caracteriza as publicações selecionadas quanto aos autores, título, periódico de publicação, tamanho da amostra e seus participantes.

**Quadro 2:** Caracterização das publicações incluídas na revisão de escopo, 2025 (N=16)

ID^*^	Autores	Título	Periódico	Amostra	Participantes
P1^†^	Nóbrega et al.^(22)^	Custo de procedimentos relacionados ao tratamento quimioterápico ambulatorial de mulheres portadoras de câncer de mama	Rev Esc Enferm USP	60 procedimentos observados em cinco etapas	Enfermeiros
P2^†^	Selwood et al.^(23)^	*Vascular access for daunorubicin during childhood acute lymphoblastic leukaemia induction treatment: a UKCCSG supportive care group and MRC childhood leukaemia working party survey*	*Eur J Oncol Nurs*	22 centros de oncologia pediátrica	Hematologistas pediátricos e profissionais de saúde
P3^†^	Silva et al.^(24)^	A visão dos enfermeiros acerca dos acessos venosos para administração da quimioterapia	Rev enferm UFPE on line	10 participantes	Enfermeiros
P4^†^	Dias et al.^(25)^	Padrões de cuidados em prevenção e tratamento de extravasamento de antineoplásicos baseado em evidências clínicas	Rev Enf Atual	30 participantes	Estudos que abordavam extravasamento e infiltração de antineoplásicos
P5^†^	Fernandes et al.^(26)^	Mulheres mastectomizadas em vigência de quimioterapia adjuvante: assistência do enfermeiro	Perspectivas Online: Biológicas & Saúde	30 participantes	Mulheres mastectomizadas entre 30 e 45 anos
P6^†^	Passos^(27)^	Validação de um sistema de classificação de pacientes para a prestação de cuidados de enfermagem em ambulatório de oncologia	Repositório Iscte Instituto Universitário de Lisboa	7 participantes	Enfermeiros
P7^†^	Freitas^(28)^	Estratégias para administração segura de antineoplásicos	Repositório Institucional Universidade Estadual Paulista	30 participantes	Enfermeiros
P8^†^	Nakamura et al.^(29)^	Avaliação dos cuidados de enfermagem em uma unidade de quimioterapia	Revista HCPA. Porto Alegre	144 procedimentos realizados por profissionais	Enfermeiros
P9^†^	Costa^(30)^	Assistência da equipe de enfermagem a pacientes oncológicos em uso de cateteres: uma revisão integrativa	Repositório Digital - URI Erechim	7 artigos	Produções científicas que abordam a assistência de enfermagem a pacientes oncológicos em uso de cateteres
P10^†^	Rodrigues^(31)^	Fatores de risco para trauma vascular durante a quimioterapia antineoplásica: contribuições do emprego do risco relativo^*^	Acta Paul. de Enferm	30 participantes	Pacientes submetidos ao tratamento quimioterápico para câncer de mama
P11^†^	Oslon et al.^(32)^	*Intravenous therapy needle choices in ambulatory cancer patients*	*Clin Nurs Res*	100 participantes	Pacientes em tratamento antineoplásico
P12^†^	Shotkin et al.^(33)^	*Use of an indwelling peripheral catheter for 3-5 day chemotherapy administration in the outpatient setting.*	*J Intraven Nurs*	89 participantes	Pacientes recebendo tratamento antineoplásico de 3 a 5 dias
P13^†^	Reis et al.^(34)^	Efeitos adversos identificados em local de infusão intravenosa periférica por drogas quimioterápicas	*Ciencia y EnfermerIa XIV*	15 participantes	Pacientes em tratamento antineoplásico por via periférica
P14^†^	Perez Fidalgo et al.^(35)^	*Management of chemotherapy extravasation: ESMO-* *EONS Clinical Practice Guidelines*	*European* *Journal of Oncology Nursing*	Não se aplica.	*Guideline*
P15^†^	Anvisa^**^ ^(36)^	Práticas seguras para a prevenção de incidentes envolvendo cateter intravenoso periférico em serviços de saúde	Agência Nacional de Vigilância Sanitária	Não se aplica.	Nota técnica
P16^†^	Gorski, LA. et al.^(37)^	*Infusion therapy standards of practice*	*Journal of Infusion Nursing*	Não se aplica.	*Guideline*

* ID - Identificação;

† P - Publicação;

** Agência Nacional de Vigilância Sanitária.

* *CIVP - Cateterismo Intravenoso Periférico.*

As publicações selecionadas apresentaram diversas práticas de enfermagem voltadas ao uso do CIVP para TA em ambulatórios^(22-37)^. Essas práticas foram organizadas em nove categorias: responsabilidade do procedimento, prevenção de infecção e biossegurança, seleção do sítio de punção, seleção do dispositivo de punção, preparo da pele, punção venosa, manutenção do acesso, cobertura e estabilização, e retirada e descarte, conforme apresentado no Quadro 3.

**Quadro 3:** Descrição das práticas de enfermagem relacionadas à TA ambulatorial com cateterismo intravenoso periférico, 2025

Categoria	Práticas de enfermagem
Responsabilidade do procedimento^(22-24)^	- Ser executado por enfermeiros ou técnicos de enfermagem.
Prevenção de infecção e biosseguranç^a(31,36^)	- Higienizar as mãos antes e após o procedimento; - Separar e utilizar os equipamentos de proteção individual.
Seleção do sítio de punção^(31,34-37)^	- Identificar um membro sem restrições, não correspondente à linfadenectomia, sem fístula arteriovenosa, sem edemas, que não apresente hemiplegia ou hemiparesia; - Evitar membros inferiores, preferindo os superiores; - Selecionar uma veia calibrosa, palpável, não tortuosa, idealmente localizada em regiões que oferecem melhor proteção a articulações; evitando-se regiões próximas a tendões, nervos e que cause menor prejuízo anatômico, caso ocorra extravasamento; - Seguir uma ordem preferencial para punção: antebraço, dorso da mão, punho e fossa ante cubital.
Seleção do dispositivo de punção^(23,24,27-36)^	- Selecionar dispositivo agulhado ou não agulhado para punção; - Selecionar um dispositivo de punção compatível com a veia escolhida, de menor calibre possível, que seja capaz de suportar a infusão da terapia.
Preparo da pele^(23,25,27,33,34-37)^	- Realizar fricção da pele com solução a base de álcool (gliconato de clorexidina a 0,5%, PVP-I^*^ alcoólico 10% ou álcool a 70%) na técnica adequada para cada solução utilizada; - Aguardar a secagem espontânea; - Não tocar no sítio de punção após assepsia da pele.
Punção venosa^(24,25,31,34-37)^	- Garrotear o membro a ser puncionado em tempo inferior a dois minutos; - Utilizar a angulação de 15º do dispositivo para a punção; - Limitar as tentativas de punção em até duas, por profissional, e cinco por paciente.
Manutenção do acesso^(25,26,29,31,34-37)^	- Checar o fluxo e refluxo imediatamente após a punção e rotineiramente antes da administração das drogas, durante e ao término; - Supervisionar a infusão de drogas vesicantes; - Irrigar a veia entre medicações e ao término da infusão com solução fisiológica ou glicofisiológica; - Monitorar constantemente o local de inserção do cateter para verificar precocemente sinais de complicação e necessidade de troca do dispositivo.
Cobertura e estabilização^(23,27,31,32,34-37)^	- Utilizar cobertura estéril, que permita a visualização do óstio, garantindo segurança e estabilização do dispositivo.
Retirada e descarte^(23,29,33,35-37)^	- Descartar imediatamente a agulha do dispositivo não agulhado e o dispositivo agulhado em um coletor de perfurocortantes; - Descartar imediatamente o cateter flexível sem desconectá-lo do circuito de infusão em um lixo químico.

* *PVP-I - Iodopovidona.*

A Figura 2 apresenta o mapa de complicações relacionadas ao uso do CIVP. Sobre as complicações associadas ao vaso destaca-se flebite^(22,29,31,32)^, esclerose venosa^(22,34)^, vasoespasmo^(31)^, tromboflebite^(31)^ e formação de cordão palpável^(32)^. No que tange à pele e ao sistema tegumentar, foram identificados edema^(25,31)^, hematoma^(28,34)^, lesão ulcerada^(22,31)^, hipercromia de pele^(28)^ e urticária^(22)^. As complicações identificadas no local de punção foram dor^(22,24,25,28,30,32)^, ardência^(24,27,34)^ e celulite^(31)^. As associadas à infusão, foram citadas extravasamento^(23,24,32,34,35)^, infiltração^(29,31)^ e infecção da corrente sanguínea^(29-32)^. A obstrução foi a única complicação decorrente do uso do dispositivo^(27)^.

**Figura 2:** Mapa das complicações relacionadas ao Cateterismo Intravenoso Periférico utilizado para Terapia Antineoplásica em ambulatórios, 2025

## DISCUSSÃO

Este estudo mapeou as evidências disponíveis na literatura sobre as práticas de enfermagem voltadas à TA utilizando o CIVP no ambiente ambulatorial e as complicações encontradas pelo uso do dispositivo, apresentando uma síntese das evidências que foram categorizadas.

A predominância de estudos descritivos entre as publicações reflete uma limitação na produção de pesquisas mais robustas, como ensaios clínicos e estudos longitudinais. Embora essas publicações forneçam um panorama geral das práticas, elas não aprofundam as análises sobre a eficácia de intervenções recomendadas, o que poderia contribuir para consolidar melhores evidências científicas na prevenção e manejo de complicações do CIVP.

A análise dos achados não evidenciou mudanças significativas nas práticas de enfermagem e nas complicações associadas ao CIVP para TA ambulatorial no período pós-COVID-19, uma vez que apenas três publicações foram recuperadas entre 2020 e 2023, sem abordagens comparativas diretas. A Nota Técnica GVIMS/GGTES/ANVISA nº 04/2022 reforça diretrizes para a segurança no uso do CIVP, enquanto o *Infusion Therapy Standards of Practice*, publicado em 2021, traz padrões internacionais para a terapia infusional, e uma revisão integrativa sintetiza evidências, mas nenhum desses documentos avalia transformações específicas antes e depois da pandemia^(30,36,37)^. No entanto, é plausível considerar que esse período fortaleceu medidas de biossegurança, o uso de equipamentos de proteção individual e a necessidade de qualificação profissional, o que sugere a importância de estudos futuros que verifiquem possíveis impactos na incidência de complicações, adesão a protocolos e capacitação dos profissionais de enfermagem.

Dois estudos abordaram a responsabilidade pela execução do procedimento. A pesquisa realizada por P2 atribui essa função a enfermeiros e técnicos de enfermagem^(22,23)^. No entanto, o estudo de P3 descreve que apenas enfermeiros são os responsáveis pela punção^(24)^. Em nível nacional, não foi identificada uma Resolução do Conselho Federal de Enfermagem (COFEN) que defina especificamente a responsabilidade pelo procedimento de punção venosa periférica. Contudo, um parecer emitido pelo Conselho Regional de Enfermagem do Estado de São Paulo (COREN-SP) indica que o procedimento pode ser realizado pela equipe de enfermagem, sendo responsabilidade do enfermeiro avaliar a condição venosa do paciente antes da realização do procedimento. O parecer ainda recomenda que, em casos de acesso venoso considerado difícil, o procedimento seja executado preferencialmente pelo enfermeiro^(38)^.

Em relação ao manejo do CIVP no ambiente ambulatorial, os estudos destacaram diversas práticas de enfermagem. A higiene das mãos foi descrita como a principal estratégia de biossegurança e prevenção das infecções relacionadas à assistência à saúde. Embora não tenha sido demonstrada a adesão dos profissionais à técnica adequada, destaca-se o estudo de P10, que elucida essa medida preventiva como uma prática de cuidado no cateter periférico, a ser realizada e monitorada frequentemente pela equipe de enfermagem^(31)^. O procedimento deve ser realizado sempre que houver a manipulação do dispositivo, seja antes, durante e/ou após. A técnica adequada pode variar em duração, conforme o método utilizado: entre 40 e 60 segundos quando feita com água e sabonete líquido, e entre 20 e 30 segundos quando realizada com preparação alcoólica^(39)^.

Quanto à escolha do local de punção, foram identificadas ações focadas na avaliação criteriosa do membro, visando selecionar um vaso adequado. Os estudos P10 e P13 ressaltam a importância de evitar membros com restrições, como aqueles afetados por linfadenectomia, fístula arteriovenosa, edema, hemiplegia ou hemiparesia, e sempre preferir os membros superiores^(31,34-37)^. Esses dados estão de acordo com um estudo brasileiro, que não observou punções em áreas com pele infectada, rompida, inflamada ou próximas a regiões ósseas e de flexão, e recomendou cateterizações nos membros superiores para prevenir complicações^(40)^.

Após a identificação do membro adequado para a punção, o estudo P4 destacou a seleção de uma veia calibrosa, palpável e não tortuosa como critérios principais^(24,34-37)^. O estudo P10 corrobora essa abordagem, recomendando veias localizadas em áreas que protejam articulações, evitando regiões próximas a tendões e nervos, além de minimizar o risco de danos anatômicos em caso de extravasamento^(31)^. Embora não haja consenso sobre o local ideal, o estudo P13 sugere o uso de veias no antebraço e dorso da mão^(34)^. No entanto, algumas áreas devem ser evitadas, pois observou-se o uso de veias de difícil visualização, tortuosas e não palpáveis^(30)^, além de punção em membro inferior em paciente adulto^(34)^.

Nesse contexto, as recomendações dos *guidelines* mais atuais vão ao encontro das práticas observadas em relação à escolha do membro e do vaso a ser puncionado. As veias recomendadas para a inserção do CIVP em adultos incluem as localizadas nas superfícies dorsal e ventral dos antebraços, como as veias cefálica, basílica, medianas do antebraço, cotovelo e dorso da mão, em membros superiores que não apresentem complicações que possam comprometer a segurança da infusão da terapia prescrita^(36,37)^.

Quanto à escolha do dispositivo a ser inserido no vaso, três pesquisas citaram a utilização de cateteres agulhados e não agulhados, sem especificação de calibre^(23,24,29)^. O estudo de P13 utilizou cateteres agulhados de calibres 21, 23 e 25 e cateteres não agulhados de calibre 24^(34)^. Já outros pesquisadores especificaram o uso de cateter *vialon* de calibres 20, 22 e 24^(33)^. Nesse sentido, um ponto de observação refere-se à necessidade de evitar o uso de cateteres agulhados e não agulhados de plástico^(32)^, visto que a literatura indica a utilização do cateter não agulhado composto de material inerte, não trombogênico, flexível, radiopaco ou transparente, de menor calibre possível^(28)^.

O estudo de P4 aponta a prática de selecionar um dispositivo de punção compatível com a veia escolhida^(25)^, e outros pesquisadores afirmam que, além da compatibilidade, existe a preocupação em escolher o menor calibre possível que suporte a infusão da terapia^(30)^. Nessa perspectiva, para melhor visualização do vaso, dois estudos relatam o uso de garroteamento do membro para ingurgitamento do vaso^(27,31)^. Corroborando, a pesquisa de P13 especifica que o garroteamento do membro é utilizado e que o tempo de permanência do garrote deve ser inferior a dois minutos, fato este a ser avaliado pela equipe de enfermagem para evitar hemólise e aumento da pressão intravascular^(34)^.

Diversos estudos ressaltam a importância de selecionar o CIVP com base no objetivo pretendido, na duração da terapia, na viscosidade dos fluidos e nas condições de acesso venoso. Cateteres de menor calibre estão associados a menor risco de flebite mecânica e química. Recomenda-se, neste caso, a utilização de cateteres flexíveis, evitando o uso de cânulas de aço, que apresentam maior risco de transfixar o vaso e causar extravasamento de medicamentos^(35-37,41)^.

Em relação ao preparo da pele para a punção, os estudos de P2, P6 e P13 descrevem o uso de preparação alcoólica a 70%^(23,27,34)^, cuja recomendação, após a aplicação da solução, é aguardar a secagem espontânea antes de iniciar a punção^(27)^. O estudo de P11 descreve a assepsia com iodo povidine a 10%, seguido de álcool a 70%, que posteriormente é seco com gaze estéril^(32)^. Associado ao preparo adequado da pele, P4 aponta a prática de não palpar o sítio de punção após a antissepsia e durante o procedimento de punção^(25)^.

O preparo da pele é indicado para a prevenção de flebite bacteriana e infecções relacionadas ao uso do dispositivo. As soluções identificadas nas práticas de preparo da pele foram semelhantes às recomendadas pela literatura, porém é importante considerar que, se for identificada sujidade visível, é necessário utilizar água e sabão ou clorexidina a 2% antes de proceder com a assepsia à base de solução alcoólica. Independentemente do antisséptico utilizado, deve-se aguardar a secagem espontânea antes de realizar a punção e não tocar novamente no local^(36,37)^.

Pesquisa descreve a angulação do dispositivo para a punção, indicando um ângulo de 15º de distância entre o bisel da agulha do cateter e a pele do paciente^(23)^. Em casos de tentativas malsucedidas, o estudo de P6 foi o único que abordou, nesse contexto, a prática de limite de tentativas de punção, sendo duas por cada profissional e cinco no total por paciente^(27)^. Outro ponto de vista refere-se ao estudo de P3, que afirma não existir limitação de tentativas até a obtenção do CIVP, mas ressalta que a angulação e a técnica adequada devem ser seguidas pela equipe de enfermagem^(24)^. Contudo, é recomendado o uso de um novo cateter a cada tentativa de punção, pois a reutilização pode levar à perda da lubrificação, obstrução e aumento do risco de complicações^(36,40)^.

Nos estudos incluídos nesta revisão, não foi identificada a prática do uso de tecnologias de visualização para punção venosa. A realização de tentativas às cegas, sem o auxílio dessas tecnologias, pode aumentar o número de tentativas necessárias para obter um acesso satisfatório, o que pode levar à depleção da rede venosa periférica^(42)^. A literatura atual recomenda o uso de metodologias como ultrassom, dispositivos de luz visível e luz infravermelha próxima em pacientes com rede venosa difícil ou após tentativas de punção sem sucesso, uma vez que essas tecnologias têm sido associadas a um maior sucesso na primeira tentativa de inserção do CIVP^(36,37,43)^.

Logo após a punção, o estudo de P4 descreve a prática de checagem de fluxo e refluxo apenas antes de iniciar a infusão da TA^(25)^. Além da checagem da perviedade do acesso antes de iniciar o antineoplásico, pesquisadores incluem mais dois momentos de testagem: durante a infusão e ao término da terapia^(29)^. Outra publicação menciona a verificação de fluxo e refluxo durante a infusão^(34)^, demonstrando uma prática difundida entre os três estudos, realizados em diferentes períodos.

Para a cobertura do CIVP, o estudo de P2 cita o uso de materiais transparentes estéreis ou fita microporosa^(23)^, enquanto P10 destaca apenas a cobertura transparente, sem especificar a esterilidade^(31)^. É importante ressaltar que o estudo de P11 traz diferentes tipos de cobertura, dependendo do tipo de cateter: os cateteres flexíveis são cobertos com curativo oclusivo e os cateteres agulhados com fita de nylon^(32)^. Em outra perspectiva, o estudo de P6, publicado recentemente em comparação aos mencionados nesta discussão, descreve a prática de seleção da cobertura, optando por aquelas que ofereçam estabilização, sejam estéreis, permitam a visualização do óstio e ofereçam segurança^(27)^.

Alguns achados corroboram um estudo que indica a predominância do uso de coberturas estéreis para cateteres venosos periféricos^(44)^. No entanto, práticas de uso de coberturas não estéreis foram identificadas em dois estudos, o que não é recomendado devido ao risco de contaminação por microrganismos. Para a cobertura do cateter periférico, o ideal é utilizar uma cobertura estéril com gaze e fita adesiva ou membrana transparente^(36,37)^.

Os estudos de P1 e P2 citam a vigilância durante a administração das drogas antineoplásicas, reforçando o monitoramento constante das drogas consideradas vesicantes, administradas através do CIVP^(22,23)^. Por esse motivo, as práticas de manutenção do dispositivo durante a infusão foram voltadas para a prevenção de complicações e o reconhecimento precoce, caso elas ocorram.

Sobre a irrigação da rede venosa, os estudos de P2 e P4 apontam a prática de lavagem do acesso venoso com solução fisiológica a 0,9% entre as medicações e ao término da TA^(23,25)^. Embora o volume a ser utilizado não tenha sido um consenso entre os estudos avaliados, é importante ressaltar que a pesquisa de P2 sugere a utilização de 50 ml da solução para essa prática^(23)^. Explorando outras soluções indicadas, uma pesquisa mantém a solução fisiológica a 0,9% ou água destilada entre as drogas e ao término^(29)^, e outra publicação (P13) acrescenta a solução glicofisiológica antes e após os antineoplásicos, ambas sem especificação de volume^(34)^.

Conforme ocorra a utilização do CIVP em nível ambulatorial, pesquisadores (P5 e P10) enfatizam a prática de monitoramento constante do local de inserção do cateter para verificar precocemente sinais de complicação. Nesse contexto, destacam a necessidade de troca do dispositivo em casos de sinais flogísticos e demais agravos^(26,31)^.

Em relação à retirada e descarte do CIVP, o estudo de P2 descreve que, no uso de dispositivo agulhado, o descarte é imediato após o término da infusão, em um coletor de perfurocortantes destinado a resíduo químico. Enquanto isso, no uso dos cateteres não agulhados, o descarte da agulha ocorre imediatamente após a punção, em um coletor de perfurocortante de risco infectante. Ao término da infusão, todo o circuito é descartado no saco plástico destinado ao resíduo do grupo B, considerado risco químico^(23)^. A pesquisa de P8 desencoraja a ocorrência de algumas situações identificadas em serviços de saúde, ilustrando que o descarte não ocorria imediatamente após a retirada do acesso venoso, deixando o dispositivo de punção solto, pendurado no suporte de soro, próximo ao local do paciente e de outros profissionais^(29)^.

No que tange ao período de troca do dispositivo, foi identificado apenas o estudo de P12, que relatou a prática de manter o CIVP do paciente de três a cinco dias para aqueles que compareciam ao ambulatório em dias subsequentes para a infusão. Assim, sugere-se, para sua manutenção, a administração do *lock* de 2 ml de heparina diariamente^(33)^. As diretrizes atuais estão em consonância com essas práticas, reforçando a segurança e eficácia na manutenção do dispositivo^(36)^.

Alguns aspectos importantes acerca das práticas de cuidados gerais com o CIVP foram identificados na pesquisa elaborada por P10. Indica-se evitar o excesso de acessórios conectados ao circuito de infusão, pois isso pode sobrecarregar o dispositivo intravenoso, bem como evitar a infusão de drogas vesicantes em bomba de infusão contínua em via periférica. Uma recomendação importante é evitar a infusão prolongada (por um tempo superior a 30 minutos) de drogas vesicantes pelo dispositivo e não utilizar veias puncionadas há mais de 24 horas^(31)^.

Sobre as complicações associadas ao uso do CIVP, as mais frequentes foram flebite, infiltração e extravasamento. Oclusão ou deslocamento do cateter e dor também são complicações bastante relatadas. Por fim, alguns agravos associados a quadros mais preocupantes incluem infecção local, infecção de corrente sanguínea associada ao cateter e extravasamento, principalmente de antraciclinas, cuja lesão tecidual é mais grave^(22,24-26,28,30,32)^.

Por meio de uma análise dos estudos que relataram as complicações, afirma-se que a notificação desses agravos associados à administração de TA ambulatorial através do CIVP é fundamental para analisar os incidentes e eventos adversos. Essa prática permite gerenciá-los e mitigá-los no que tange à sua ocorrência, implementando práticas baseadas em evidências científicas e substituindo aquelas obsoletas e consideradas de risco para a segurança do procedimento^(28)^. Um estudo de revisão sistemática, realizado em 2023, corrobora e reforça que a notificação das complicações é fundamental para o gerenciamento de riscos assistenciais que comprometem a segurança do paciente^(45)^.

### Limitações do estudo

Em relação às limitações do estudo, a restrição de descritores voltados à temática em nível ambulatorial deve ser considerada, o que pode ter excluído estudos durante a estratégia de busca.

### Contribuições para a Área

Este estudo poderá contribuir para o corpo de conhecimento sobre as práticas de cuidado com o CIVP para TA realizada ambulatorialmente, bem como suas complicações associadas. Embora a revisão de escopo não seja utilizada para a implementação de evidências, ressalta-se que os resultados poderão contribuir para as ações de cuidado ao paciente, especialmente àqueles que utilizam a atenção secundária para resolver sua demanda decorrente do tratamento oncológico.

## CONCLUSÕES

Este estudo permitiu mapear, na literatura, as práticas de cateterismo intravenoso periférico para a administração da TA ambulatorial, bem como as complicações associadas ao uso do dispositivo. Foram identificadas práticas de enfermagem que poderão orientar ações nos serviços de saúde, possibilitando a discussão dos achados disseminados na literatura. Essas práticas incluem: responsabilidade pelo procedimento, prevenção de infecção e biossegurança, seleção do sítio de punção, escolha do dispositivo de punção, preparo da pele, punção venosa, manutenção do acesso, cobertura e estabilização, e retirada e descarte do dispositivo.

Além dessas práticas, destacam-se complicações como flebite, edema, dor, ardência, extravasamento e infecção da corrente sanguínea. Esta pesquisa apresentou o estado da arte, utilizando a metodologia de revisão de escopo, e servirá como um guia para a assistência ao paciente, promovendo a resolução de demandas relacionadas aos tratamentos oncológicos na atenção secundária.

## Data Availability

Os dados da pesquisa estão disponíveis no corpo do artigo.
